# The Prevention of Cholera

**Published:** 1896-09

**Authors:** 


### THE PREVENTION OF CHOLERA.

The beneficial effects of careful sanitary inspection and precautions against cholera are well brought out in the recent report of the medical officer of the British Local Government Board. The history of the cholera epidemics of 1848 and 1853 which reached England by the northern route, through Russia, North Germany, and the North Sea ports, was in each case that of a severe outbreak in the year following its arrival. Thus in 1848 1,105 persons died of cholera and the next year 53,293; in 1853 there were 4,419 cholera deaths, and in 1854 no fewer than 20,097. In 1892 cholera arrived in Germany by the same route, but the British government sent out a number of special medical officers who carefully investigated the sanitary condition of the country, paying special attention to the seaports, and with sufficient powers for the suppression of nuisances. Owing to the precautions taken no case occurred in England in 1892, but there were 135 deaths in 1893, a small number when we consider the greatly increased source of infection in these times of close international communication. It was feared that 1894 would see a considerable outbreak, but on the contrary, owing to the extraordinary care taken, not one death took place in that year. From the financial point of view Dr. Thorne-Thorne has been able to prove that the value of the life saved more than outweighs the expense incurred.

